# Safe to Spare? Predictors of Oncological Safety for Nerve-Sparing Technique during Robot-Assisted Radical Prostatectomy in High-Risk Prostate Cancer. Insight from a High-Volume Center with Centralized mpMRI Review

**DOI:** 10.1590/S1677-5538.IBJU.2025.0413

**Published:** 2026-02-20

**Authors:** Luca Lambertini, Fabrizio Di Maida, Giulia Carli, Antonio Andrea Grosso, Sofia Giudici, Anna Cadenar, Simone Sforza, Daniele Paganelli, Filippo Lipparini, Neliana Kucuku, Rossella Catanzaro, Francesca Conte, Francesco Lupo Conte, Matteo Salvi, Simone Agostini, Fausto De Nisco, Gabriella Nesi, Rino Oriti, Gianni Vittori, Andrea Minervini, Andrea Mari

**Affiliations:** 1 University of Florence Careggi Hospital Department of Experimental and Clinical Medicine Florence Italy Department of Experimental and Clinical Medicine, University of Florence - Unit of Urology and Andrology, Careggi Hospital, Florence, Italy; 2 Azienda Ospedaliero-Universitaria Careggi Department of Radiology Firenze Italy Department of Radiology, Azienda Ospedaliero-Universitaria Careggi, Firenze, Italy; 3 University of Florence Department of Health Sciences, Division of Pathological Anatomy Florence Italy Department of Health Sciences, Division of Pathological Anatomy, University of Florence, Florence, Italy

**Keywords:** Methods, Robotic Surgical Procedures, Prostatic Neoplasms

## Abstract

**Objective:**

To evaluate patterns and imaging-based predictors of positive surgical margins (PSMs) in patients with high-risk prostate cancer (PCa) undergoing nerve-sparing (NS) robot-assisted radical prostatectomy (RARP).

**Materials and Methods:**

We retrospectively analyzed 1,235 consecutive patients with high-risk PCa treated with RARP between 2022 and 2024 at a high-volume tertiary referral center. Among them, 533 patients underwent preoperative multiparametric MRI (mpMRI) reviewed by two expert uro-radiologists and MRI–ultrasound fusion biopsy. A per-side analysis was performed to identify predictors of ipsilateral PSMs in cases where neurovascular bundle (NVB) preservation was attempted. Biochemical recurrence (BCR) was assessed, and multivariable logistic regression was used to determine independent predictors of PSMs.

**Results:**

Overall, 36.1% of patients underwent non–nerve-sparing surgery, 49.5% unilateral NS, and 14.4% bilateral NS. Nerve sparing was performed on 418 surgical sides, with ipsilateral PSMs detected in 90 (21.5%). Sides with and without PSMs showed comparable nerve-sparing techniques (intra- vs interfascial) and similar 3-year BCR-free survival rates (68% vs. 69%) at a median follow-up of 36 months, although earlier biochemical failure at lower PSA thresholds was more frequent in PSM-positive sides. On multivariable analysis, larger prostate volume, apical tumor location, peripheral zone involvement, greater lesion diameter, and extracapsular extension on mpMRI were independently associated with an increased risk of ipsilateral PSMs.

**Conclusions:**

Nerve-sparing RARP may be feasible in carefully selected high-risk PCa patients. The integration of mpMRI-based predictors can enhance patient selection, optimizing the balance between oncologic safety and functional preservation. Prospective studies are warranted to validate these findings and minimize selection bias.

## INTRODUCTION

During the constant evolution of urologic surgery, the primary objective of radical prostatectomy (RP) has always been to eradicate prostate cancer (PCa) while striving to maintain the functionality of the pelvic organs whenever feasible (1). In this scenario, a nerve-sparing (NS) technique plays a key role in erectile function and might positively affect postoperative urinary continence (2), also potentially leading to positive surgical margins (PSM) and, therefore, biochemical recurrence (BCR) (3). Currently, the European Association of Urology (EAU) guidelines do not define strong recommendations over neurovascular bundle (NVB) preservation, only suggesting avoiding NS surgery when there is a clinical risk of ipsilateral extra-capsular extension (ECE) based on clinical staging, biopsy Gleason score or multiparametric magnetic resonance imaging (mpMRI) (4). To date, few large retrospective studies failed to confirm significant correlations between NVB preservation and BCR, risk of metastasis or mortality (5), even in high-risk patients with a baseline PSA over 20 ng/mL (6). These findings are possibly driven by several specific advancements in the urological surgery panorama. Particularly, Robot-assisted RP (RARP) enables us to perform a meticulous nerve-sparing also minimizing positive surgical margins (PSM), with a short learning curve supporting its global adoption (7). Indeed, the surgeon experience remains critical to minimizing PSM, particularly during nerve-sparing procedures where oncologic control and functional preservation must be carefully balanced (8). Moreover, a careful assessment of the MRI images also allows surgeons to better forecast and avoid potential areas of ECE, thereby reducing the likelihood of PSM (9). Three-dimensional models offer enhanced tumor and gland visualization, yet their application remains largely confined to research and has not been widely adopted in routine clinical practice (10).

The feasibility of nerve-sparing approaches in borderline cases, such as those involving high-risk (HR) patients, remains underexplored with limited studies addressing this critical aspect of surgical decision-making. An incremental NS technique, tailored according to the risk group, has been proposed for RARP (11). However, the direct impact of this approach on PSM and BCR remains unclear.

The aim of this study was to identify predictors of ipsilateral positive surgical margins in patients undergoing NS-RARP for high-risk PCa in a novel per-side fashion, based on side-specific mpMRI features in selected patients treated with RARP for HR-PCa in a tertiary referral institution, where the diagnostic process, including mpMRI and fusion biopsy, was centralized.

## MATERIALS AND METHODS

### Study Design

After Institutional Review Board approval, we retrospectively analyzed a consecutive cohort of patients treated with robot-assisted radical prostatectomy (RARP) for high-risk prostate cancer (PCa) between January 2020 and April 2023 at our tertiary referral center. Among this overall surgical population, a predefined subset of patients underwent preoperative multiparametric magnetic resonance imaging (mpMRI) performed and centrally reviewed at our institution by two expert uro-radiologists, as well as MRI–ultrasound fusion biopsy. The present study and all subsequent per-side analyses were restricted to this mpMRI-reviewed subgroup, which constituted the analytical cohort for side-specific evaluation of nerve-sparing decisions and ipsilateral positive surgical margins.

Main inclusion criteria were as follows: 1) high-risk PCa according to the EAU risk groups for biochemical recurrence (4); 2) mpMRI performed and evaluated or re-evaluated by two expert uro-radiologists (S.A., F.D.N.) at our Center; 3) MRI-ultrasound fusion biopsy performed by expert urologists or uro-radiologists at our center or; 4) having completed a minimum of 24 months follow-up.

Main exclusion criteria were as follows: 1) Previous therapy for PCa, including RT, focal therapy or androgen deprivation therapy (ADT); 2) Evidence of distant metastasis, including non-regional lymph nodes, at preoperative conventional staging, consisting in bone scintigraphy (BS) and computed tomography (CT), or PET-PSMA. These exams were performed in all included patients; 3) mpMRI or ultrasound fusion biopsy performed in another center or performed at our center by other operators with limited expertise.

Included patients were stratified according to the preservation of bundle: radical (Group A), monolateral nerve-sparing (NS) (Group B), bilateral NS (Group C).

### Surgical technique

All procedures were performed by four experienced surgeons (≥100 RARP), with a standard six ports transperitoneal configuration. The Da Vinci surgical system Si, X or Xi (Intuitive Surgical, Sunnyvale, CA, USA) were used throughout the cases. In case of anticoagulant/antiplatelet (AC/AP) therapy, its suspension, replacement or maintenance was agreed with the anesthesiologist and the patient. An anterograde approach was chosen in all cases. Concomitant standard pelvic lymph node dissection (PLND) was performed in all cases according to standard anatomical landmarks, including the removal of lymphatic tissue along the external iliac vessels, within the obturator fossa, and around the internal iliac vessels. NS was performed with an intrafascial dissection between the peri-prostatic veins and the pseudocapsule of the prostate, or interfascial dissection along the peri-venous plane; or extrafascial dissection.

According to demographic, clinical and pathological features, NS side and technique were performed according to surgeon preference, also discussed and agreed with the patient prior to the surgery. The decision to perform NS or radical dissection was based on surgeon discretion and intraoperative judgement, which inevitably introduces selection bias. This limitation was acknowledged and considered in statistical interpretation. Change of strategy of NS technique during treatment was also reported. Pelvic drainage was placed at the end of the procedure at surgeon's discretion. A 18Ch Foley catheter was inserted during the procedure in all cases. Catheter removal was scheduled according to the surgeon's judgment.

### Covariates and outcomes

The following clinical, imaging, and pathological variables were prospectively collected: patient age, body mass index (BMI), Charlson Comorbidity Index (CCI), presence of hypertension or diabetes mellitus, smoking status (categorized as current, former, or never smoker), history of previous transurethral resection of the prostate (TURP), and previous major abdominal surgery. Preoperative evaluation included serum prostate-specific antigen (PSA) levels and findings on digital rectal examination (DRE) performed by the attending urologist. Multiparametric MRI (mpMRI) parameters included prostate volume, Prostate Imaging Reporting and Data System (PI-RADS) score of the dominant lesion, maximum diameter of the index lesion, anatomical location (apex, mid-gland, base), and zonal distribution (anterior, peripheral, transitional zones). Radiological features suggestive of ECE and SVI were systematically assessed, with ECE defined by the presence of neurovascular bundle asymmetry, capsular bulge or irregularity, measurable extracapsular disease, or obliteration of the recto-prostatic angle on high-resolution T2-weighted images, and SVI based on direct tumor extension into seminal vesicles, low signal intensity, or abnormal contrast enhancement. In the absence of these features, organ-confined disease was assumed. Biopsy variables included ISUP grade group assignment according to the modified Gleason scoring system endorsed by the 2005 and 2014 ISUP consensus conferences, and clinical staging (cT and cN) were determined based on DRE and imaging findings. PSA persistence was defined as a prostate-specific antigen level ≥0.1 ng/mL measured at the first postoperative assessment, 4–6 weeks after surgery. Biochemical failure (BCF) was defined as the first occurrence of PSA ≥0.1 ng/mL at any time during follow-up, thereby capturing early biochemical progression and necessarily including patients with PSA persistence. Biochemical recurrence (BCR) was defined as a PSA ≥0.2 ng/mL confirmed on two consecutive measurements, in accordance with European Association of Urology guidelines, and was used to estimate biochemical recurrence–free survival. BCF and BCR represent distinct, non–mutually exclusive oncologic endpoints evaluated at different PSA thresholds and with different confirmation requirements; therefore, their proportions are not expected to sum to 100%. UI was defined as the use of >1 pad per day. ED was defined as an IIEF-5 score <12 (moderate or severe).

### Follow-up

First postoperative assessment included catheter removal, Kegel exercises were routinely illustrated concomitantly. Therefore, a postoperative assessment was scheduled approximately 30 days after surgery, and included clinical, histopathological and laboratory evaluation. Patients interested in erectile function recovery who underwent NS-RARP were provided with PDE5i treatment, which was offered free of charge by the National Health System (12) The International Index of Erectile Function-5 (IIEF-5) was self-administered to assess sexual function. Therefore, patients were followed up at 3, 6 and 12 months postoperatively, and then twice a year. Total PSA, urinary incontinence (UI) and erectile disfunction (ED) were evaluated.

## Statistical analysis

Continuous variables are presented as median (IQR: interquartile range) and differences between groups were tested by Student's independent t-test or Mann-Whitney U-test according to their normal or non-normal distribution, respectively (normality of variables’ distribution was tested by Kolmogorov-Smirnov test). Proportional data were assessed using Pearson's Chi-square test. Per-patient and per-side analyses were performed to evaluate PSM rate. Oncologic outcomes were evaluated on a per-patient analysis. Univariate and multivariate logistic regression analyses (MVA) were performed to identify independent predictors for clinically meaningful outcomes. Candidate variables included PSA, DRE findings, clinical T stage, and all mpMRI-derived features. Collinearity diagnostics were assessed using Variance Inflation Factors (VIF > 5) and Spearman's correlation. PSA and DRE were found to be highly correlated with MRI-derived markers (ECE and lesion diameter) and clinical T stage (VIFs = 4.8–5.1), leading to their exclusion from the final model to prevent multicollinearity. A sensitivity multivariable model including PSA and DRE is presented in Supplementary Table-1. Statistical significance was set as p <0.05. Of note, all subsequent per-side analyses were conducted exclusively within the subgroup of patients who underwent centralized mpMRI review and fusion biopsy at our institution, as detailed in the Methods section. Statistical analysis was performed using SPSS v. 27 (IBM SPSS Statistics for Mac, Armonk, NY, IBM Corp).

## RESULTS

Overall, 533 patients were included in the study. Median patient age was 69 years (IQR: 64–73), and median Charlson Comorbidity Index (CCI) was 3 (IQR: 2–4). Median body mass index (BMI) was 25.7 kg/m² (IQR: 23.9–27.8). Most patients had hypertension (72%) or diabetes mellitus (63%), with fewer having undergone previous major abdominal surgery (18%) or previous endoscopic treatment for benign prostatic obstruction (6.6%). Median baseline PSA was 7.4 ng/mL (IQR: 5.4–11), and DRE was positive in 82% of cases. RARP for HR PCa was performed either radically in 192 (36.1%), or with monolateral or bilateral NS in 264 (49.5%) and 77 (14.4%) patients, respectively (Supplementay Table-2). By dividing patients for the type of NS approach, a higher median age recorded in the radical group (p=0.01) was found compared to the Monolateral and Bilateral NS groups. Moreover, the radical group showed a significative higher rate of positive DRE and of clinical ECE at mpMRI (p=0.01 and p=0.02, respectively) compared to the counterparts. Baseline comorbidity burden, assessed by the Charlson Comorbidity Index and the prevalence of hypertension and diabetes mellitus, was comparable across nerve-sparing groups and is detailed in Table-1. For interpretability, CCI was also categorized as 0–2, 3–4, and ≥5. Functional outcomes (continence and erectile recovery) were stratified according to NS status, showing superior recovery in NS patients, although baseline functional status may have influenced surgical choice. These differences underscore the presence of selection bias, as patients with more adverse clinical features were more often managed with a radical approach. At per-side analysis, nerve-sparing was applied to 418 sides during RARP, with PSM identified in 90 (21.5%) of these sides. Sides with PSM exhibited larger median lesion diameters on mpMRI compared to those free of PSM (18 mm [IQR: 10–22] vs. 12 mm [IQR: 10–16], p=0.01) and a greater frequency of lesions >15 mm (44% vs. 27%, p=0.02). Additionally, PIRADS 5 lesions were more prevalent on sides with PSM compared to sides without (44% vs. 24%, p=0.02). Apical lesion location was markedly more frequent in sides with PSM (58% vs. 26%, p=0.01), while mid (19% vs. 31.7%) and basal locations (23% vs. 42.3%) were less common (p=0.01). The presence of extracapsular extension (ECE) (44% vs. 27%, p=0.001) and seminal vesicle invasion (SVI) (21% vs. 12%, p=0.01) at mpMRI were higher in sides harboring PSM. Histological assessment confirmed pathological ECE was more frequent among sides with PSM (75% vs. 53%, p=0.01). Other relevant MRI and biopsy characteristics are detailed in Table-2.

**Table 1:** Baseline, clinical and pathological preoperative features of patients with High risk PCa treated with Robot Assisted Radical Prostatectomy (RARP).

Characteristics		Overall N = 533^1^	Bilateral Radical N = 1921	Monolateral NS N = 264^1^	Bilateral NS N = 77^2^	p-value^2^
Age years, median (IQR)		73 (66-77)	75 (69-79)	73 (64-76)	67 (62-71)	**0.04**
BMI (kg/m2), median (IQR)		25.7 (23.9-27.8)	26.3 (22.1-29.9)	23.8 (21.6-27.6)	28.1 (24.2-31.2)	0.3
**CCI, median (IQR)**						0.8
	0-2	229 (43)	89 (46)	109 (42)	31(41)	
	3-4	298 (56)	101 (52)	152 (57)	45 (58)	
	≥5	6 (1)	2 (2)	3 (1)	1 (1)	
Hypertension, n (%)		384 (72%)	142 (74%)	187 (71%)	55 (71%)	0.6
Diabetes mellitus, n (%)		336 (63%)	120 (63%)	171 (64%)	45 (61%)	0.7
Previous endoscopic surgery for BPO, n (%)		35 (6.6%)	13 (6.7%)	18(6.8%)	4 (5.1)	0.2
Previous major abdominal surgery, n (%)		95 (18%)	35 (18%)	44 (16%)	16 (20%)	0.12
Preoperative PSA, median (IQR)		7.4 (5.4-11)	9.1 (6.7-13.2)	7.2 (6.1-10)	5.9 (4.6-9)	0.08
Positive DRE, n (%)		437 (82%)	184 (95%)	213 (81%)	40 (52%)	**0.01**
Prostate Volume (cc), median (IQR)		52 (38-67)	54 (34-63)	49 (33-72)	42 (28-58)	0.18
Main lesion dimension at MRI, median (IQR)		19 (12-18)	21 (16-29)	18 (10-22)	9 (7-14)	**0.01**
Extracapsular estension, n (%)		202 (38%)	95 (49%)	88 (33%)	19 (24%)	**0.01**
Seminal Vescicles Invasion, n (%)		64 (12%)	26 (13.5%)	32 (12%)	6 (7.7)	0.06

BMI = Body Mass Index; BPO = Benign Prostatic Obstruction; CCI = Charlson Comorbidity Index, PSA = Prostate Specific Antigen; DRE = Digito-Rectal Examination

1 Values are reported as median (interquartile range) or number (percentage), as appropriate.

2 P-values were calculated using Mann–Whitney U test, Student's t-test, or Chi-square test, as appropriate.

**Table 2:** Magnetic Resonance Imaging (MRI) and pathological preoperative features of patients with High risk PCa treated with Nerve Sparing Robot Assisted Radical Prostatectomy (RARP).

Characteristic		Overall N = 418^1^	NO PSM same side N = 328^1^	PSM same side N = 90^2^	p-value^2^
Prostate Volume (cc), median (IQR)		45 (33-59)	48 (34-63)	45 (33-72)	0.13
Prostate Volume >80cc, n(%)		54 (13%)	34 (10.4%)	20 (22.2%)	**0.01**
**PIRADS 5** **(same side of bundle preservation), n (%)**					**0.02**
	Yes	118 (28%)	78 (24%)	40 (44%)	
**PIRADS >15mm** **(same side of bundle preservation), n (%)**					**0.02**
	Yes	127 (30%)	88 (27%)	39 (44%)	
Main lesion dimension at MRI, median (IQR)		14 (12-18)	12 (10-16)	18 (10-22)	**0.01**
**PIRADS location, n (%)**					**0.01**
	Apex	137 (33%)	85 (26%)	52 (58%)	
	Mid	121 (29%)	104 (31.7%)	17 (19%)	
	Base	160 (38%)	139 (42.3%)	21 (23%)	
**PIRADS zone lesion, n (%)**					**0.001**
	Anterior	21 (5%)	14 (4.3%)	7 (8%)	
	Periferic	322 (77%)	267 (76%)	55 (61%)	
	Transitional	75 (18%)	47 (19%)	28 (31%)	
Extracapsular estension, n (%)		129 (31%)	89 (27%)	40 (44%)	**0.001**
Seminal Vescicles Invasion, n(%)		18 (9%)	7 (21%)	11 (12%)	**0.01**
**ISUP grade, at biopsy, n (%)**					0.06
	4	374 (89%)	296 (90%)	78 (86%)	
	5	44 (11%)	32 (10%)	12 (14%)	
**Clinical T stage, n(%)**					0.2
	cT1c	79 (19%)	62 (19%)	17 (18%)	
	cT2a	80 (19%)	65 (20%)	15 (16%)	
	cT2b	9 (2%)	7 (2%)	2 (2%)	
	cT2c	183(44%)	134 (41%)	49 (51%)	
	cT3a	58 (14%)	46 (14%)	12 (13%)	
	cT3b	9 (2.1%)	8 (2.2%)	1 (2%)	
**cN, n(%)**					0.6
	cN0	391 (93%)	307 (94%)	82 (91%)	
	cN1	27 (7%)	21 (6%)	8 (9%)	

1 Values are reported as median (interquartile range) or number (percentage), as appropriate.

2 P-values were calculated using Mann–Whitney U test, Student's t-test, or Chi-square test, as appropriate.

Regarding perioperative outcomes, median operative time was 165 minutes (IQR: 124–193), with slightly longer operative durations observed for sides with PSM compared to those without (183 vs. 161 minutes, p=0.08). An intraoperative switch of nerve-sparing technique was recorded in 9.1% of cases, occurring more frequently in sides subsequently harboring positive surgical margins (21.0% vs. 6.1%, p=0.015), with the most common modification being from an intrafascial to an interfascial dissection.

Pathological staging demonstrated that pT3a tumors occurred more frequently in sides with PSM compared to those without (75% vs. 53%, p=0.01), while the rates of pT2 and pT3b stages were lower or comparable (Table-3). Among nerve-sparing techniques, interfascial dissection was the most adopted approach (85%), whereas intrafascial dissection was used in 15% of cases, with no significant difference according to PSM occurrence (p=0.08). Biochemical failure (PSA ≥0.1 ng/mL during follow-up) occurred in 211/418 sides (50.4%), with a slightly shorter median time to biochemical failure in sides with PSM (4 months [IQR: 1–9]) compared with those without PSM (6 months [IQR: 3–12], p=0.07), consistent with Table-3. In terms of 3-year biochemical-recurrence-free survival, no significant differences were observed between sides without and with PSM (68% vs 69%, p=0.8). At multivariable analysis, larger prostate volume (OR: 1.02; 95% CI: 1.01–1.03; p=0.01) and PIRADS lesion diameter (OR: 1.01; 95% CI: 1.006–1.32; p=0.02), apical lesion location (OR: 2.03; 95% CI: 1.23–3.37; p=0.01), peripheral vs transitional zone lesion (OR: 3.22; 95% CI: 1.18–4.76; p=0.001) and ECE (OR: 4.19; 95% CI: 2.89–6.86; p=0.001) detected at MRI were independently associated with the presence of PSM. In a sensitivity model including PSA and DRE, neither variable was independently associated with ipsilateral PSM (PSA p = 0.27; DRE p = 0.18), and model discrimination (AUC = 0.80) was comparable to the primary mpMRI-based model (AUC = 0.79), confirming the robustness of our findings. Other factors, including ISUP grade 5 on target biopsy and intraoperative switch of nerve-sparing technique, were not significantly associated with positive surgical margins (Table-4).

**Table 3:** Perioperative, pathological and follow-up features of patients with high risk PCa treated with RARP with neurovascular bundle preservation.

Characteristic		Overall N = 418^1^	NO PSM same side N = 328^1^	PSM same side N = 90^1^	p-value^2^
**Operative Time (min),** median (IQR)		165 (124-193)	161 (111-181)	183 (158-203)	0.08
**Estimated blood loss (mL),** median (IQR)		200 (100- 350)	200 (80-300)	220 (120-400)	0.2
**Intraoperative Complications,** n. (%)		6 (1.3%)	4 (1.2%)	2 (2.2)	0.12
**Intraoperative switch of NS technique,** n %					0.014
	from intrafascial to interfascial NS	32 (7.7%)	15 (4.6%)	17 (18.8%)	
	from intrafascial to extrafascial NS	5 (1.2%)	4 (1.2%)	1 (1.1%)	
	from interfascial to extrafascial NS	1 (0.2%)	1 (0.3%)	0 (0%)	
**Nerve sparing technique performed,** n. (%)					0.08
	Intrafascial	84 (20.1%)	46 (14%)	17 (18.8%)	
	Interfascial	328 (78.5%)	266 (81.1%)	62 (68.9%)	
	Extrafascial	6 (1.4%)	5 (1.5%)	1 (1.1%)	
**Time to drainage removal (days),** median (IQR)		1(1-2)	1(1-2)	1(1-2)	0.9
**Length of stay (days),** median IQR		3 (2-4)	3 (2-4)	3 (2-4)	0.9
**Pathological T stage,** n (%)					0.01
	pT2	64 (15%)	54 (17%)	10 (11%)	
	pT3a	235 (57%)	174 (53%)	61 (75%)	
	pT3b	111 (27%)	92 (29%)	19 (21%)	
**Pathological N stage,** n. (%)					0.2
	pN0	384 (92%)	302 (92%)	77 (86%)	
	pN1	34 (8%)	26 (8%)	13 (14%)	
**PSA persistence,** n. (%)		17 (4.1%)	12 (3.7%)	5 (5.5%)	0.11
**Biochemical failure,** n. (%)		211 (50.4%)	165 (50.3%)	46 (51.1%)	0.09
**3 Years Biochemical Recurrence Free Survival,** n (%)		284 (68%)	222 (68%)	62 (69%)	0.8
**Time to BCF (months)**		6 (1-12)	6 (3-12)	4 (1-9)	0.07
**Follow up (months),** median (IQR)		36 (24-40)	36 (24-40)	32 (20-40)	0.08

Min = minutes; IQR = Inter Quartile Range; BCF = Biochemical failure

1 Values are reported as median (interquartile range) or number (percentage), as appropriate.

2 P-values were calculated using Mann–Whitney U test, Student's t-test, or Chi-square test, as appropriate.

**Table 4:** Multivariable analysis assessing the clinical predictors of positive surgical margin on the same side of neurovascular bundle preservation after Robot-Assisted Radical Prostatectomy for High-Risk Prostate Cancer.

Covariates		OR	95%CI	p value
Prostate Volume (100cc)		1.02	1.01-1.03	0.01
**PIRADS location**				
	Base	Ref		
	Mid	1.34	0.72 – 2.45	0.2
	Apex	2.03	1.23 – 3.37	0.01
**PIRADS zone lesion**				
	Anterior	Ref		
	Peripheral	3.22	1.18 – 4.76	0.001
	Transitional	1.8	0.85 – 2.73	0.4
	PIRADS dimension (mm)	1.01	1.006 - 1.32	0.02
**ISUP >4 on target samples**				
	No	Ref		
	Yes	1.38	0.94-1.88	0.06
**Extracapsular estension at MRI**				
	No	Ref		
	Yes	4.19	2.89-6.86	0.001
Intraoperative switch of NS technique (yes/no)		1.26	0.94-1.67	0.11

OR = Odds Ratio; CI = Confidence Interval; MRI = Magnetic Resonance Imaging

## DISCUSSION

Preoperative mpMRI has been shown to impact surgical planning by identifying laterality and extent of disease, allowing refinement of the NS approach (13). The NS surgery in HR-PCa remains a debated strategy due to the increased risk of ECE and PSM (14). However, advances in preoperative imaging and the precision of RARP have broadened the indications for NS in selected patients (15, 16). The EAU guidelines do not contraindicate NS in HR-PCa but recommend avoiding it when clinical or radiological predictors of ECE are present (4). The novelty of our work lies in the per-side analytical approach with centralized mpMRI review, allowing for refined correlation between imaging predictors and ipsilateral PSM.

High-quality evidence supporting the safety of NS in HR-PCa remains limited, particularly in standardized cohorts with centralized imaging and pathology review (17, 18). As a result, most available data on NS originates from low- or intermediate-risk populations, where the oncologic risk is lower and functional preservation is more routinely prioritized (19). In contrast, the application of NS in HR-PCa is yet to be determined, despite growing interest in surgical de-escalation strategies. Evidence specific to HR-PCa, and particularly to side-specific nerve preservation, is scarce. However, HR-PCa is a heterogeneous entity, and in selected patients with unilaterally localized lesions, wide bilateral excision may be unnecessarily aggressive (20, 21). When imaging confirms unilateral disease without features suggesting ECE, unilateral or incremental NS may be a feasible compromise. An mpMRI-based nomogram has been developed to guide NS grade selection based on the probability of ECE, offering a side-specific, risk-adapted approach to optimize the balance between functional recovery and oncologic control (22).

In this study, we evaluated the postoperative and oncological outcomes of NS-RARP in a highly selected cohort of patients with HR-PCa, treated in a tertiary referral center with centralized preoperative staging using mpMRI and fusion-targeted biopsy. All patients fulfilled high-risk criteria according to EAU classification, including ISUP grade group ≥4 (63.8%) or PSA >20 ng/mL (23.8%), while 48.5% had clinical stage ≥T3a. Nerve preservation was planned based on side-specific radiological and pathological findings, allowing risk-adapted surgical planning. Bilateral nerve-sparing was initially intended in 18.8% of cases, unilateral in 57.9%, and no nerve-sparing in 23.2%, with final decisions refined intraoperatively according to local findings.

From an oncologic standpoint, approximately half of the cohort experienced biochemical failure at the 0.1 ng/mL threshold during follow-up, and about one-third developed BCR within 3 years, highlighting that in carefully selected patients, nerve-sparing did not worsen short-term oncologic outcomes compared with non-nerve-sparing approaches, whereas long-term oncologic equivalence remains uncertain given the limited follow-up. This apparent discrepancy reflects the different biological and temporal meaning of the two endpoints, as BCF captures early PSA progression at a lower threshold, whereas BCR represents a more stringent and confirmed definition of biochemical relapse.

We also evaluated the role of different NS techniques—classified as intrafascial, interfascial, and extrafascial—performed on each side in relation to mpMRI features and pathological outcomes. The planned dissection plane was defined preoperatively but frequently modified intraoperatively based on direct assessment. These changes were systematically recorded, allowing us to analyze how surgical adaptability influenced ipsilateral PSM rates in relation to preoperative imaging and tumor localization. In our series, interfascial dissection was the most frequently performed nerve-sparing technique, representing 56.2% of all sides. Intrafascial dissection was used in 25.2% of cases, while a radical approach (i.e., complete excision of the bundle) was chosen in 18.6%, particularly when intraoperative findings raised concern for extracapsular extension. Notably, in 21.7% of cases, the initial nerve-sparing strategy was modified intraoperatively, most often by downgrading the dissection plane based on intraoperative judgment. No significant difference in PSM was observed between interfascial and intrafascial approaches, supporting the effectiveness of preoperative planning and intraoperative adaptability. This approach mirrors the logic of graded nerve-sparing, where the dissection plane is tailored according to side-specific oncologic risk and is consistent with EAU recommendations to avoid nerve preservation only in the presence of clear predictors of extracapsular disease.

To better evaluate the relationship between nerve-sparing and surgical margins, we moved from a per-patient to a per-side analytical framework, allowing for a more granular assessment of how side-specific tumor features and surgical decisions influence ipsilateral PSM.

In this setting, our first key finding was that several mpMRI features were independently associated with the occurrence of PSM on the same side of NS, thus jeopardizing the oncological safety of surgery. Particularly, the presence of larger prostate volume (p=0.04), higher PIRADS lesion diameter (p=0.02), apical lesion location (p=0.01) as well as peripheral vs transitional zone lesion (p=0.001) predicted the occurrence of positive margins, also when the procedure was carried out by a highly experienced robotic surgeon. These findings potentially represent a strong argument in favor of the widening of surgical indications for the NS adoption, thus highlighting the importance of tailored surgical planning when balancing functional and oncologic outcomes(23-26). To date, no clinical features were included in the preoperative PSM prediction. In contrast, several series proposed a validated nomogram-based model incorporating mpMRI findings—including PI-RADS score, lesion location, and capsular contact—alongside clinical parameters. Particularly, Soeterik et al. (24) developed a preoperative risk model that combines mpMRI and clinical variables to predict side-specific ECE, with posterior and lateral lesion location identified as important contributors to risk stratification. Similarly, Nyarangi-Dix et al. proposed a nomogram that integrates imaging features such as PI-RADS score and lesion localization to improve prediction accuracy for ECE in high-risk patients (25, 27-30). Ostau et al. externally validated a bicenter risk model emphasizing the role of mpMRI markers—including lesion location and extent of capsular contact—for anticipating ECE, demonstrating improved performance over clinical-only tools (26). Although these models are primarily designed to predict ECE rather than PSMs directly, the anatomical predictors they incorporate—particularly posterior and apical involvement—are consistent with our findings. These regions are known to pose technical challenges during prostatectomy, and their accurate delineation on mpMRI is critical for margin control.

Our study further emphasizes the utility of centralized imaging review, which likely enhanced the consistency and reliability of lesion localization and feature interpretation. On the other hand, the centralization of the radiologic assessment enhanced the statistical predictive value of MRI predictors over the clinical one, particularly when only a high-risk patient's subset is analyzed. Indeed, while we did not use nomograms, our results underscore the pivotal role of high-quality, anatomically detailed mpMRI in predicting adverse pathological outcomes and guiding intraoperative strategy, particularly in clinical settings where predictive models are not routinely applied. An additional consideration concerns the generalizability of our findings. The predictive value of the identified variables relies heavily on high-quality mpMRI acquisition and expert interpretation, which were ensured in this study through centralized imaging review by dedicated uro-radiologists. Consequently, the applicability of our results may be limited in settings with heterogeneous imaging protocols or limited radiologic expertise. External validation in multicenter cohorts with varying levels of mpMRI standardization is therefore warranted before broader clinical implementation.

The present manuscript is not devoid from limitations. Firstly, the retrospective study design and the single center fashion might have induced several non-negligible biases in the analysis. Nevertheless, the centralization of the imaging assessment represented a key factor to properly evaluate the overall burden of preoperative imaging-based surgical planning on patient's outcomes. Secondly, the relatively small sample size might lower the replicability of the reported results. Moreover, the lack of randomization might have overestimated the NS feasibility in more complex cases. The absence of a statistically significant association between ISUP 5 and positive surgical margins in the multivariable model might reflect collinearity between histologic grade and MRI-detected extracapsular extension, both representing closely interrelated indicators of tumor aggressiveness. When both variables were included, ECE emerged as the stronger anatomical predictor, attenuating the apparent effect of ISUP grade. Similarly, although PSA and DRE are established clinical parameters in most preoperative risk models, in our cohort both were strongly correlated with MRI-derived markers of tumor extent, resulting in multicollinearity and their subsequent exclusion from the final model. Sensitivity analyses confirmed that their inclusion did not enhance model discrimination, reinforcing the predominance of imaging-derived predictors in this high-risk, imaging-centralized population. Nevertheless, it should be noted that the limited number of events per variable (90 sides with PSM) may have reduced the statistical power to detect weaker independent effects of clinical variables. With larger cohorts and a higher event count, modest associations for PSA, DRE, or ISUP grade could potentially emerge as significant. Key limitation is the potential for selection bias in NS choice. Importantly, our findings should be interpreted in the context of radiotherapy plus ADT being a well-established standard of care in HR-PCa, and surgery with NS may be considered only in individualized cases. Furthermore, the follow-up period of 36 months is too short to draw definitive oncologic conclusions in HR-PCa. The omission of key predictors such as PSA and DRE from final models further limits clinical generalizability.

## CONCLUSIONS

The adoption of a nerve-sparing approach during RARP represents a feasible option only in carefully selected high-risk prostate cancer patients. Accordingly, the identification of imaging-based predictors such as prostate volume, apical location, peripheral zone involvement, lesion size, and ECE might enhance the patient selection process, thus optimizing the balance between functional and oncologic outcomes. These aspects warrant further evaluation in randomized settings and may help guide clinical decision-making.

## Data Availability

All data generated or analysed during this study are included in this published article

## References

[1] Wang J, Hu K, Wang Y, Wu Y, Bao E, Wang J (2023). Robot-assisted versus open radical prostatectomy: a systematic review and meta-analysis of prospective studies. J Robot Surg.

[2] Michl U, Tennstedt P, Feldmeier L, Mandel P, Oh SJ, Ahyai S (2016). Nerve-sparing surgery technique, not the preservation of the neurovascular bundles, leads to improved long-term continence rates after radical prostatectomy. Eur Urol.

[3] Wang X, Wu Y, Guo J, Chen H, Weng X, Liu X (2019). Oncological safety of intrafascial nerve-sparing radical prostatectomy compared with conventional process: a pooled review and meta-regression analysis. BMC Urol.

[4] Cornford P, van den Bergh RCN, Briers E, Van den Broeck T, Brunckhorst O, Darraugh J (2024). EAU-EANM-ESTRO-ESUR-ISUP-SIOG guidelines on prostate cancer 2024 update. Part I: screening, diagnosis, and local treatment with curative intent. Eur Urol.

[5] Preisser F, Gandaglia G, Arad F, Karakiewicz PI, Bandini M, Pompe RS (2021). Association of neurovascular bundle preservation with oncological outcomes in patients with high-risk prostate cancer. Prostate Cancer Prostatic Dis.

[6] Spirito L, Chessa F, Hagman A, Lantz A, Celentano G, Sanchez-Salas R (2024). Long-term oncological outcomes after nerve-sparing robot-assisted radical prostatectomy for high-risk localized prostate cancer. Diagnostics (Basel).

[7] Bonet X, Moschovas MC, Onol FF, Bhat KR, Rogers T, Ogaya-Pinies G (2021). The surgical learning curve for salvage robot-assisted radical prostatectomy: a prospective single-surgeon study. Minerva Urol Nephrol.

[8] Da Cruz JAS, Porto BC, Terada BD, Gonçalves FGA, Orra SH, Martinez JVN (2025). Does the surgeon's learning curve impact pentafecta outcomes in radical prostatectomy? a systematic review and meta-analysis. BMC Urol.

[9] Schiavina R, Bianchi L, Borghesi M, Dababneh H, Chessa F, Pultrone CV (2018). MRI displays the prostatic cancer anatomy and improves bundle management before robot-assisted radical prostatectomy. J Endourol.

[10] Checcucci E, Pecoraro A, Amparore D, De Cillis S, Granato S, Volpi G (2022). The impact of 3D models on positive surgical margins after robot-assisted radical prostatectomy. World J Urol.

[11] Martini A, Cumarasamy S, Haines KG, Tewari AK (2019). An updated approach to incremental nerve sparing for robot-assisted radical prostatectomy. BJU Int.

[12] Siena G, Mari A, Canale A, Mondaini N, Chindemi A, Greco I (2018). Sexual rehabilitation after nerve-sparing radical prostatectomy: free-of-charge phosphodiesterase type 5 inhibitor administration improves compliance. J Sex Med.

[13] Panebianco V, Salciccia S, Cattarino S, Minisola F, Gentilucci A, Alfarone A (2012). Use of multiparametric MR with neurovascular bundle evaluation to optimize oncological and functional management in nerve-sparing radical prostatectomy. J Sex Med.

[14] Bejrananda T, Takahara K, Sowanthip D, Motonaga T, Yagi K, Nakamura W (2023). Comparing pentafecta outcomes between nerve sparing and non nerve sparing robot-assisted radical prostatectomy in a propensity score-matched study. Sci Rep.

[15] Day E, Tzelves L, Dickinson L, Shaw G, Tandogdu Z (2025). Impact of preoperative surgical planning in robotic-assisted radical prostatectomy on trifecta outcomes: systematic review and meta-analysis. Minerva Urol Nephrol.

[16] Hu A, Lin Y, Zhu X, Li J, Luo F, Yu X (2025). Does transurethral resection of the prostate before robot-assisted radical prostatectomy have adverse effects on patients diagnosed with prostate cancer: a comparative evidence-based analysis?. J Robot Surg.

[17] Morozov A, Barret E, Veneziano D, Grigoryan V, Salomon G, Fokin I (2021). Nerve-sparing surgery for high-risk prostate cancer: a systematic review. Minerva Urol Nephrol.

[18] Ditonno F, Bologna E, Licari LC, Franco A, Cannoletta D, Checcucci E (2025). Neurovascular structure-adjacent frozen-section examination (NeuroSAFE) during robot-assisted radical prostatectomy: a systematic review and meta-analysis of comparative studies. Prostate Cancer Prostatic Dis.

[19] Liu Y, Deng XZ, Qin J, Wen Z, Jiang Y, Huang J (2023). Erectile function, urinary continence and oncologic outcomes of neurovascular bundle sparing robot-assisted radical prostatectomy for high-risk prostate cancer: A systematic review and meta-analysis. Front Oncol.

[20] Kumar A, Samavedi S, Bates AS, Mouraviev V, Coelho RF, Rocco B (2017). Safety of selective nerve sparing in high-risk prostate cancer during robot-assisted radical prostatectomy. J Robot Surg.

[21] Humke C, Hoeh B, Preisser F, Wenzel M, Welte MN, Theissen L (2022). Concordance between mpMRI and pathological stage and its influence on nerve-sparing surgery. Curr Oncol.

[22] Martini A, Soeterik TFW, Haverdings H, Rahota RG, Checcucci E, De Cillis S (2022). Algorithm to personalize nerve sparing in unilateral high-risk prostate cancer. J Urol.

[23] Mod M, Güngör HS, Karaca H, Tahra A, Sobay R, İnkaya A (2025). Factors determining early continence after robotic radical prostatectomy. J Clin Med.

[24] Soeterik TFW, van Melick HHE, Dijksman LM, Küsters-Vandevelde H, Stomps S, Schoots IG (2022). Nomogram to predict side-specific extraprostatic extension. Eur Urol Oncol.

[25] Nyarangi-Dix J, Wiesenfarth M, Bonekamp D, Hitthaler B, Schütz V, Dieffenbacher S (2020). Combined clinical parameters and mpMRI for prediction of extraprostatic disease. Eur Urol Focus.

[26] Ostau NEV, Handke AE, Wiesenfarth M, Albers P, Antoch G, Noldus J (2024). Bicenter validation of a risk model for the preoperative prediction of extraprostatic extension of localized prostate cancer combining clinical and multiparametric MRI parameters. World J Urol.

[27] Martini A, Falagario UG, Villers A, Dell'Oglio P, Mazzone E, Autorino R (2020). Contemporary techniques of prostate dissection for robot-assisted prostatectomy. Eur Urol.

[28] Gamal A, Moschovas MC, Saikali S (2025). Comparing technological and intraoperative performances of da Vinci Xi and da Vinci 5 robotic platforms. Int Braz J Urol.

[29] Moschovas MC, Patel V (2022). Nerve-sparing robotic-assisted radical prostatectomy: how I do it after 15,000 cases. Int Braz J Urol.

[30] Moschovas MC, Jaber A, Saikali S (2024). Long-term functional and oncologic outcomes after RARP following USPSTF PSA recommendations. Int Braz J Urol.

