# Supplementary material for: Safe to Spare? Predictors of Oncological Safety for Nerve-Sparing Technique during Robot-Assisted Radical Prostatectomy in High-Risk Prostate Cancer. Insight from a High-Volume Center with Centralized mpMRI Review
Source: Int Braz J Urol. 2026 Feb 20;52(3):e20250413. doi: 10.1590/S1677-5538.IBJU.2025.0413 (PMC13124193; doi:10.1590/S1677-5538.IBJU.2025.0413)
Supplement: APPENDIX [file 1677-6119-ibju-52-03-e20250413-suppl1.pdf]

APPENDIX

Supplementary Table 1 - Sensitivity multivariable model accounting for both clinical and mp MRI features.

Variable	OR	95% CI	p value
**PSA (ng/mL)**	1.01	0.99–1.03	0.27
**Positive DRE**	1.34	0.86–1.94	0.18
**Prostate volume**	1.02	1.01–1.03	0.01
**Apical lesion**	2.11	1.24–3.43	0.01
**Peripheral zone lesion**	3.12	1.14–4.71	0.001
**ECE at mpMRI**	3.98	2.74–6.65	<0.001
**Model AUC**	0.80 (vs 0.79 in main model)		

Supplementary Table 2. Sensitivity multivariable model accounting for both2 - clinical and mp MRI features

Variable	OR	95% CI	p value
**PSA (ng/mL)**	1.01	0.99–1.03	0.27
**Positive DRE**	1.34	0.86–1.94	0.18
**Prostate volume**	1.02	1.01–1.03	0.01
**Apical lesion**	2.11	1.24–3.43	0.01
**Peripheral zone lesion**	3.12	1.14–4.71	0.001
**ECE at mpMRI**	3.98	2.74–6.65	<0.001
**Model AUC**	0.80 (vs 0.79 in main model)		
